# Heat-Related Deaths in Hot Cities: Estimates of Human Tolerance to High Temperature Thresholds

**DOI:** 10.3390/ijerph110303304

**Published:** 2014-03-20

**Authors:** Sharon L. Harlan, Gerardo Chowell, Shuo Yang, Diana B. Petitti, Emmanuel J. Morales Butler, Benjamin L. Ruddell, Darren M. Ruddell

**Affiliations:** 1 School of Human Evolution & Social Change, Arizona State University, Tempe, AZ 85287, USA; E-Mails: gchowell@asu.edu (G.C.); Emmanuel.J.Morales@asu.edu (E.J.M.B.); 2 School of Mathematical & Statistical Sciences, Arizona State University, Tempe, AZ 85287, USA; E-Mail: syang65@asu.edu; 3 Department of Biomedical Informatics, Arizona State University, 13212 East Shea Boulevard, Scottsdale, AZ 85259, USA; E-Mail: Diana.Petitti@asu.edu; 4 Department of Engineering and Computing Systems, Polytechnic School, Arizona State University, 330M Peralta Hall, Mesa, AZ 85212, USA; E-Mail: bruddell@asu.edu; 5 Spatial Sciences Institute, University of Southern California, 3616 Trousdale Parkway, AHF B55, Los Angeles, CA 90089, USA; E-Mail: druddell@usc.edu

**Keywords:** apparent temperature, climate, gender, heat-related deaths, hot climate, hot cities, temperature threshold

## Abstract

In this study we characterized the relationship between temperature and mortality in central Arizona desert cities that have an extremely hot climate. Relationships between daily maximum apparent temperature (AT_max_) and mortality for eight condition-specific causes and all-cause deaths were modeled for all residents and separately for males and females ages <65 and ≥65 during the months May–October for years 2000–2008. The most robust relationship was between AT_max_ on day of death and mortality from direct exposure to high environmental heat. For this condition-specific cause of death, the heat thresholds in all gender and age groups (AT_max_ = 90–97 °F; 32.2‒36.1 °C) were below local median seasonal temperatures in the study period (AT_max_ = 99.5 °F; 37.5 °C). Heat threshold was defined as AT_max _at which the mortality ratio begins an exponential upward trend. Thresholds were identified in younger and older females for cardiac disease/stroke mortality (AT_max_ = 106 and 108 °F; 41.1 and 42.2 °C) with a one-day lag. Thresholds were also identified for mortality from respiratory diseases in older people (AT_max_ = 109 °F; 42.8 °C) and for all-cause mortality in females (AT_max_ = 107 °F; 41.7 °C) and males <65 years (AT_max_ = 102 °F; 38.9 °C). Heat-related mortality in a region that has already made some adaptations to predictable periods of extremely high temperatures suggests that more extensive and targeted heat-adaptation plans for climate change are needed in cities worldwide.

## Introduction

Deaths from exposure to extreme heat have been studied in cities on all inhabited continents [1,2,3,4,5,6,7]. Motivating this research are high numbers of deaths during extreme heat events and projected increases in deaths from heat-related causes due to climate change [8,9]. Heat waves in all types of climate zones cause many deaths in cities during short-term atypical weather. In cities with tropical and subtropical climates, hot weather also lasts for long stretches during a prolonged warm season each year, leading to chronic heat exposure in the population [10,11]. Normal daily temperatures in summer are predictably high and hours of exposure to high temperatures in a 24-hour period are also longer compared to temperate cities.

Information about heat-related deaths in hot cities is important to hundreds of millions of people who live in them and also contributes to understanding the limits of human tolerance to climatic conditions that may become more prevalent worldwide in this century. Average air temperatures in most cities are becoming warmer over time for two different reasons: global climate change and the urban heat island effect [12,13]. Current temperature-mortality relationships in hot cities are one indicator of the extent to which humans have adapted physiologically, behaviorally, and technologically to climate and offer insights about living in a future warmer world. 

The purpose of the study was to measure daily mortality response to temperatures during the warmest months in southwestern U.S. desert cities that have made adaptations to a very hot climate. Our study’s focus on enduring heat complements literature on episodic heat events that cause extraordinary numbers of deaths in a short time in cities with more temperate climates [3,5,6,7,8]. We also distinguish among causes of death in hot weather separately by age and gender. The quality of mortality data for this type of study is relatively good in the U.S. The world’s hottest cities are located in deserts of the Middle East and Africa [14], but civil registrations of deaths are low worldwide [15] and there are especially high error rates in assigning primary cause of death on death certificates in developing nations [16,17]. 

## 2. Experimental Section

### 2.1. Study Region

**Figure 1:**
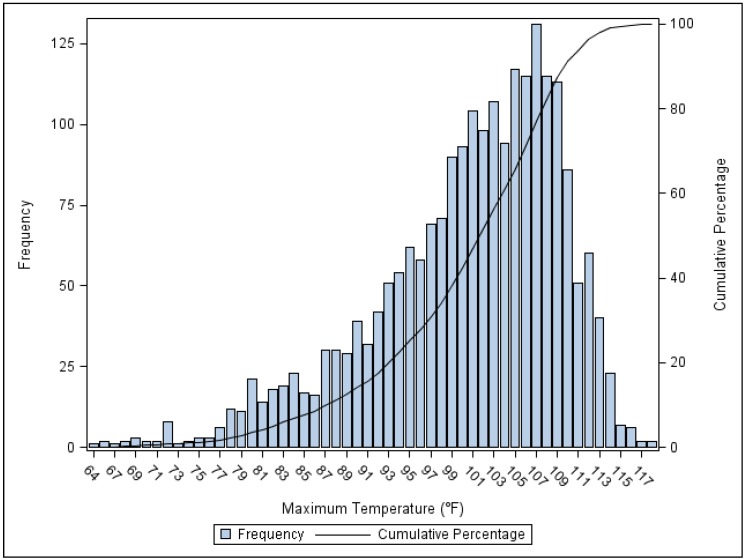
Frequency and cumulative percentage of days by maximum temperature (°F) at the Sky Harbor International Airport Weather Station during the months May–October for years 2000–2008.

Maricopa County in the Sonoran Desert of south-central Arizona comprises 60% of the state’s population and it also includes a vast expanse of wilderness (2010 population = 3.8 million, area = 9224 sq. mi.). The urbanized area of this county encompasses Phoenix (the largest city), 15 adjoining municipalities, and three Native American communities (2010 U.S. Census Urban Area polygons, total population = 3.6 million, area = 1261 sq. mi.). In the Phoenix area, the 30-year average daily maximum temperature during May–October for years 1981–2010 was 99.6 °F, which tied with Yuma, Arizona for the highest temperature of all major weather stations in the U.S. [18]. Figure 1 shows the frequencies and cumulative percentage of days by maximum temperature that occurred during our study period. This region also has multi-day summer extreme heat events, which exceed historically normal local mean temperatures and usually occur in July and less frequently in June and August [19].

Replacement of natural desert and agricultural land cover with residential, commercial, and industrial development began in the early 20th century and accelerated during the Sunbelt economic expansion from the 1970s to 2008, contributing to a strong urban heat island effect in the settled area [20,21]. Ruddell *et al.* [22] compared historical temperatures between Phoenix (urban) and Gila Bend (rural) National Weather Service (NWS) regional surface weather stations in central Arizona and found pronounced warming in Phoenix and modest warming in Gila Bend. These differences suggest that global climate change is warming natural/rural areas while the combined forces of urbanization and global change are warming urban environments more rapidly. Local and global changes in climate have increased the heat burden in the naturally hot desert and are projected to continue doing so in the 21st century [23].

### 2.2. Data

The study period covers the warm season in the Arizona desert during the months May–October for years 2000–2008. Sky Harbor International Airport in Phoenix is the only NWS first-order surface weather station in the study region (first-order stations collect a comprehensive array of weather variables every hour on a 24-hour basis). Using hourly air temperature and dew point temperature data from Sky Harbor, we calculated hourly apparent temperature (AT) for the study period. We derived daily minimum, mean, and maximum AT (AT_min, _AT_mean, _AT_max)_ from the hourly data. Although temperatures in urban environments are spatially heterogeneous due to land cover and built environment characteristics [24,25], time-series studies often use a single airport or center-city station to examine temperature-mortality relationships for a city or metropolitan area (e.g., [4,26]).

AT is a measure of how hot it feels to people, taking into account relative humidity as well as air temperature [27]. We used Equation (1) to estimate hourly AT [28,29].

AT = ‒2.653 + (0.994 × T) + (0.0153 × Tdp^2^) + wind_adj_ (1)

where T = Ambient dry-bulb temperature (°C); Tdp = dew point temperature (°C); wind_adj_ = adjustment to AT for given T and wind-speed (meters/second) [30].

AT is a good measure for this region because it accounts for human discomfort in the humid summer rainy season (July–August) and in the dry warm months. This equation overestimates AT at Tdp < 0 °C (32 °F); therefore, for days in our study period with Tdp < 0 °C (<3% of all days), we set Tdp = 0 to reduce the error in AT estimates for these days. After this substitution, our AT estimates were in close agreement with Steadman’s AT values for his temperature-humidity scale (p. 862) [27]. In our analysis, temperatures were converted to Fahrenheit (°Celsius × 9/5 + 32 = °Fahrenheit). During the study period, median AT_max _= 99.5 °F; 10% of all days < 83 °F; and 10% of all days > 109 °F. Table 1 describes average daily AT_max _during the study period by month.

**Table 1:** Summary statistics for daily maximum apparent temperatures (AT_max _°F) based on daily measurements obtained from the Sky Harbor International Airport Weather Station during the months May–October for years 2000 to 2008.

Month	AT_max_ Mean	AT_max_ SD	AT_max_ Minimum	AT_max_ Maximum
May	92	9.2	66	111
June	103	6.3	85	118
July	107	4.6	93	118
August	105	5.3	87	118
September	99	6.4	80	113
October	84	8.3	60	104

We obtained mortality data for the study period from Part I of death certificates filed in Maricopa County. For each record, age, gender, cause of death, and date of death were retrieved from the original records. Institutional Review Boards at Arizona State University and the Arizona Department of Health Services approved our use of these data for this study.

Prior literature has estimated heat-related mortality for all causes [1,2,4,5,6,8,26] and for different disease categories, most consistently cardiovascular and respiratory diseases [2,31,32,33,34]. To examine all-cause mortality related to temperature, we followed the example of others [1,5,26,35,36] and excluded most external causes of death. We excluded International Classification of Diseases, 10th Revision (ICD-10) codes S00-99, T00-66, T68-98, U00-99, V01-99, W00-99, X00-29, 31, 33-53, 55-84, Y00-98, Z00-99 and included T67.x, X30, X32 and X54 because they are heat-related. We examined eight condition-specific cause-of-death categories derived from a conceptual model of medically adverse effects of exposure to high environmental heat and prior literature on environmental heat and mortality. These condition-specific cause-of-death categories are described next. The Supplementary Material details the reasoning behind selection of categories and Supplemental Table S1 lists all ICD-10 codes by category:

*Direct exposure to environmental heat.* Conditions that might directly cause death in situations of high environmental heat were mapped to corresponding primary cause-of-death ICD-10 codes. Also included in this category were deaths with terms associated with exposure to high environmental heat (e.g., “heat exhaustion”) entered as free text in any of the four underlying cause-of-death fields of the death certificate Part 1 (Table S1).

*Dehydration*. Dehydration is a common and potentially lethal consequence of exposure to high environmental heat. The ICD-10 codes for dehydration and its direct consequence—volume depletion—and conditions indicative of dehydration (e.g., lack of water) were selected for this category. Also included were deaths with “dehydration” entered as free text in any of the cause-of-death fields (Table S1).

*Possible consequences of heat and dehydration*. This category was defined by ICD-10 codes for conditions that are possible consequences of exposure to high environmental heat and/or dehydration and terms (e.g., “hypovolemia”) entered as free text in the underlying cause-of-death fields (Table S1). Figure S1 graphically depicts the model that explains our approach to selecting codes considered likely to be due to the physiologic and pathophysiologic consequences of heat and dehydration/volume depletion/hypovolemia.

Five other condition-specific categories were selected because prior research has found elevated mortality during hot weather. We defined these categories as follows: *cardiac disease/stroke* (ICD-10 codes I20.x-I25.x, I49.9, I60.x-I79.x); *chronic renal failure* (N18.x, N19); *heart failure* (I50.x, I51.6-I51.9); *chronic obstructive pulmonary disease (COPD)/asthma* (J43.x-J45.x, J46); and *other respiratory diseases* (all other ICD-10 respiratory codes J00-J42, J47-J99.x).

Mortality at AT_max_ for each cause-specific category and all causes was calculated as the daily ratio of actual mortality to baseline May–October (“summer”) mortality defined by the equation:

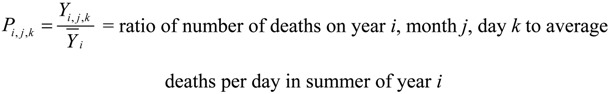
(2)
where *P_i,j,k_* = mortality ratio; *Y_i,j,k_* = number of deaths on year *i*, month *j*, day *k*; 
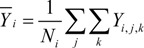
 = average deaths per day in summer of year *I*; *N_i_* = number of days in year *i* from May–October.

### 2.3. Statistical Analysis

We used cubic spline regressions with four knots (equal interval quintiles) to describe the temperature-mortality relationship for each category during the study period. The daily time series values for AT_max_, AT_mean_, and AT_min_ were highly correlated with each other. Consequently, the spline regressions for all three AT measures produced very similar results. Models for AT_max_ are presented because exposure to peak temperature is most likely during daytime. 

Mortality ratios were the response variables in Equation (3). Predictor variables were AT_max_ with 0, 1, 2, and 3-day lags as well as an indicator binary variable for heat wave days. Heat waves were defined by Meehl and Tebaldi’s method [37], which compares each study year to historical temperatures and identifies extreme heat events based on three conditions: daily maximum temperature must be above the 97.5 percentile of historically normal conditions for at least three days; average daily temperature must be above the 97.5 percentile for the entire heat event; and daily maximum temperature must be above the 81 percentile for the entire event. The binary variable for heat wave days significantly improved model fit for the categories direct exposure to heat, cardio disease/stroke, and all-cause deaths. Additional predictor variables for day of the week and public holiday were not statistically significant for any category and were eliminated from the models.

Mortality ratio (*P_i,j,k_*) = spline(AT_max-0-day-lag_) + spline(AT_max-1-day-lag_) + spline(AT_max-2-day-lag_) + spline(AT_max-3-day-lag_) + β_1_ × heat wave (3)

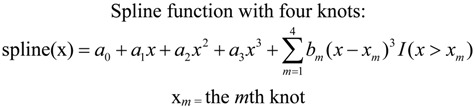


For every condition-specific cause-of-death category and all-cause deaths, we selected the temperature metric (AT_max _with 0, 1, 2 or 3 day lags) that had the largest positive coefficient and a statistically significant effect (*p* < 0.05) on increasing mortality ratio in the spline regression model. We then used Poisson regression to model the relationship between the temperature metric and mortality in order to identify high temperature thresholds. A lower bound for AT_max_ was established in the flat part of the temperature-mortality curve by inspection, and we subsequently tested candidate heat thresholds at every integer value of AT_max _(°F) above the lower bound up to the maximum observed temperature in our data (118 °F). From all candidate models, we selected the model and associated threshold that provided the best exponential fit as deemed by the goodness-of-fit statistic (Pearson chi-squared/df). Our estimate of the heat threshold is the temperature at which the mortality ratio begins an upward exponential trend. We checked our models for overdispersion and found significant overdispersion only in the category direct exposure to environmental heat. Hence, for this outcome we employed a negative binomial regression model. For each cause-of-death category in which a high temperature threshold was identified, we estimated the relative risk (RR) of death above the threshold per 1 °F. We note that it was not possible to identify a heat threshold for some of the mortality cause-specific categories when the AT_max_ was not a significant predictor of mortality.

## 3. Results and Discussion

### 3.1. Results

The statistical analysis in this study is restricted to decedents who were residents of Maricopa County. Table 2 displays, for each cause-specific category and all-cause deaths, the number of resident deaths (column 2) and the percentage of residents of all deaths that occurred in the county (column 3).

**Figure 2:**
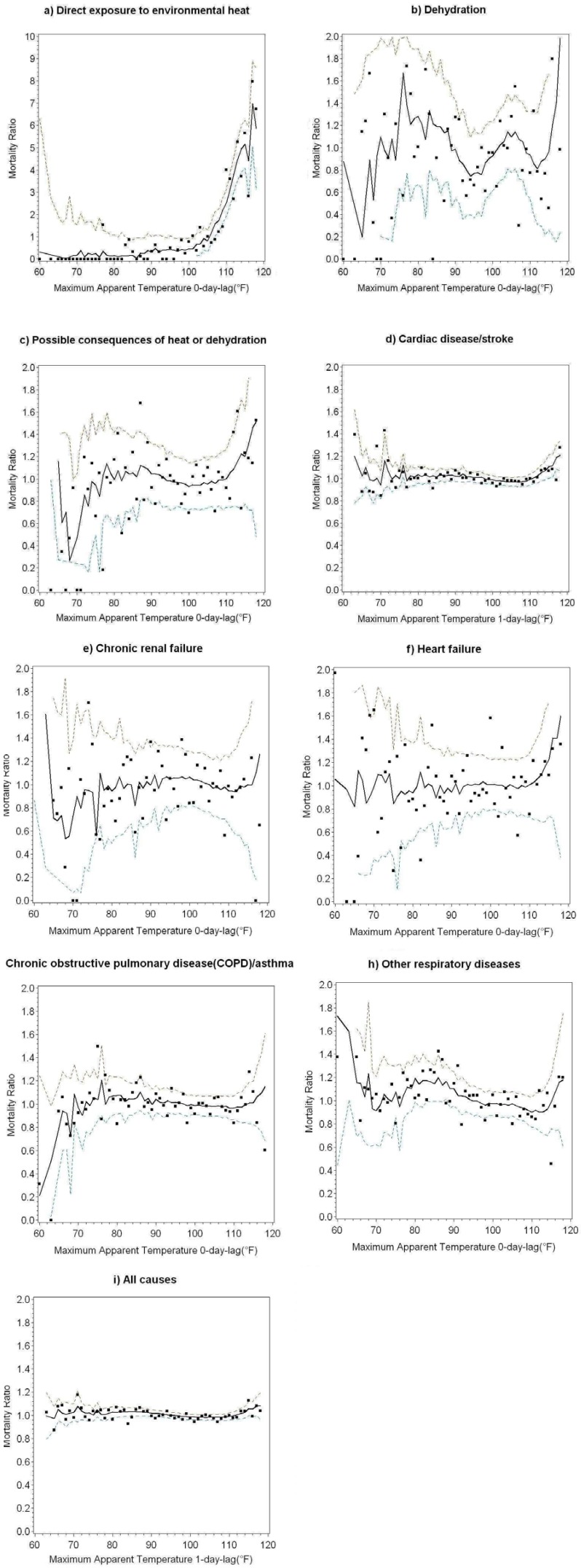
Temperature-mortality relationships for cause-specific categories and all-cause deaths for Maricopa County residents (all ages) during the months May–October for years 2000–2008. Small black squares ■ are the average mortality ratios (*P_i,j,k _*in Equation (2)) or ratio of number of actual deaths to average deaths per day aggregated for all years) at a given maximum apparent temperature (AT_max_). Values < 1.0 indicate fewer than expected deaths; values > 1.0 indicate more than expected deaths. Note the scale of mortality ratio on the y-axis in (**a**) is different from the other graphs. Solid black lines are the cubic spline regressions predicting daily average mortality ratios at AT_max_ (unsmoothed). Dashed lines are the upper and lower 95% confidence intervals (CIs).

Figure 2a–i shows the relationships between AT_max_ and mortality ratios (*P_i,j,k_* in Equation (2)) for residents using fitted cubic spline models with 95% confidence intervals (CI) for cause-specific categories and all-cause deaths. Corresponding to Figure 2, threshold temperatures and RR/CI for deaths above thresholds are reported in Table 2 for category-specific and all-cause deaths with statistically significant AT_max _coefficients (*p* < 0.05).

The strongest relationship between mortality and temperature was for direct exposure to high environmental heat on day of death (Table 2, row 1 and Figure 2a). In this category, we observed the lowest temperature threshold (AT_max_ = 93 °F, lag days = 0) and largest increase in the relative risk of death from heat exposure (RR = 1.20); *i.e.*, daily mortality increased by 20% per 1 °F above threshold. The relative risk (RR = 1.05) from possible consequences of heat or dehydration was also increased above daily same-day AT_max _≥ 106 °F. We found higher temperature thresholds, one-day lags, and smaller increases in the relative risk of death from cardiac disease/stroke (AT_max_ = 110 °F, RR = 1.03) and all-causes (AT_max_ = 107 °F, RR = 1.01) than for deaths directly attributable to heat exposure and consequences of heat or dehydration. 

To determine whether heat had different effects on population subgroups, we re-estimated mortality models separately for ages <65 years and ≥65 years (Table 3). We identified significant AT_max_ relationships with mortality ratio in both age groups in the categories direct exposure to environmental heat and cardiac disease/stroke and in all-cause deaths. For direct exposure to heat, the temperature threshold for <65 (AT_max_ = 93 °F) was 3 °F *higher* than for age ≥65 (AT_max_ = 90 °F) with zero lag days. However, for cardiac disease/stroke, the temperature threshold for age <65 (AT_max_ = 106 °F) was 3 °F *lower* than for age ≥65 (AT_max_ = 109 °F) with one-day lags. Similarly, for all-cause deaths, the temperature threshold for age <65 (AT_max_ = 104 °F) was 3 °F lower than for age ≥65 (AT_max_ = 107 °F) with one-day lags. The increases in relative risk above threshold temperatures within each category of death were very similar for both age groups. In people age ≥65, there were statistically significant effects of temperature on COPD/asthma deaths (AT_max_ = 110 °F, lag days = 0, RR = 1.05). 

**Table 2:** Deaths in Maricopa County residents directly caused by exposure to environmental heat and other causes potentially related to high temperatures during the months May–October for years 2000–2008.

Cause of Death ^a^	Number of County Resident Deaths	Percent Resident Deaths of All Deaths ^c^	Threshold AT_max _(°F) (Lag Days)^ d^	RR above Threshold ^e^	95% CI^ f^
Direct exposure to environmental heat	215	73.1	93 (0)	1.20	1.16, 1.23
Dehydration	409	93.8			
Possible consequences of heat or dehydration	1114	92.1	106 (0)	1.05	1.01, 1.09
Cardiac disease/stroke	24,717	91.2	110 (1)	1.03	1.01, 1.05
Chronic renal failure	762	89.8			
Heart failure	848	92.0			
Chronic obstructive pulmonary diseases (COPD)/asthma	5342	92.5			
Other respiratory diseases	3639	89.3			
All causes^ b^	90,284	91.4	107 (1)	1.01	1.00, 1.02

^a^ See Supplemental Material and Supplemental Table S1 for ICD-10 codes and keywords included in cause-of-death categories; ^b ^Excludes 9969 deaths of from external causes (ICD-10 codes S00-99, T00-66, T68-98, U00-99, V01-99, W00-99, X00-29, 31, 33-53, 55-84, Y00-98, Z00-99) and 187 deaths missing a county of residence address; ^c ^Number of Maricopa County residents in each cause-of-death category divided by total number of decedents in category; ^d ^Coefficient for AT_max_ (appropriate lag) has *p*-value < 0.05 in best fitting Poisson regression model (Pearson chi squared/df); ^e ^Relative risk (RR) is the effect estimate for the increase in mortality per 1 °F above the threshold temperature; ^f^ Confidence interval (CI).

**Table 3:** Deaths in Maricopa County residents ages <65 years and ≥65 years directly caused by exposure to environmental heat and other causes potentially related to high temperatures during the months May–October for years 2000–2008.

	<65 Years			≥65 Years		
Cause of Death ^a^	Threshold AT_max _(°F) (Lag Days) ^c^	RR above Threshold ^d^	95% CI ^e^	Threshold AT_max _(°F) (Lag Days)^ c^	RR above Threshold^ d^	95% CI^ e^
Direct exposure to environmental heat	93 (0)	1.19	1.15, 1.24	90 (0)	1.21	1.15, 1.26
Possible consequences of heat ordehydration	106 (0)	1.12	1.02, 1.23			
Cardiac disease/stroke	106 (1)	1.03	1.00, 1.05	109 (1)	1.03	1.01, 1.05
Chronic obstructive pulmonary diseases (COPD)/asthma				110 (0)	1.05	1.00, 1.10
All causes^ b^	104 (1)	1.01	1.00, 1.02	107 (1)	1.01	1.00, 1.02

^a ^See Supplemental Material and Supplemental Table S1 for ICD-10 codes and keywords included in cause-of-death categories; ^b ^Excludes deaths from external causes (see Table 2); ^c ^Coefficient for AT_max_ (appropriate lag) has *p*-value < 0.05 in best fitting Poisson regression model (Pearson chi squared/df); ^d ^Relative risk (RR) is the effect estimate for the increase in mortality per 1 °F above the threshold temperature; ^e^ Confidence interval (CI).

**Table 4:** Deaths in Maricopa County male residents ages <65 years and ≥65 years directly caused by exposure to environmental heat and other causes potentially related to high temperatures during the months May–October for years 2000–2008.

	Male < 65 Years			Male ≥ 65 Years		
Cause of Death ^a^	Threshold AT_max _(°F) (Lag Days)^ c^	RR above Threshold ^d^	95% CI^ e^	Threshold AT_max _(°F) (Lag Days)^ c^	RR above Threshold^ d^	95% CI ^e^
Direct exposure to environmental heat	92 (0)	1.19	1.15, 1.24	90 (0)	1.21	1.14, 1.29
Possible consequences of heat or dehydration	106 (0)	1.18	1.06, 1.33			
Other respiratory diseases				109 (0)	1.09	1.01, 1.16
All causes^ b^	102 (1)	1.01	1.00, 1.02			

^a ^See Supplemental Material and Supplemental Table S1 for ICD-10 codes and keywords included in cause-of-death categories; ^b ^Excludes deaths from external causes (see Table 2); ^c ^Coefficient for AT_max_ (appropriate lag) has *p*-value < 0.05 in best fitting Poisson regression model (Pearson chi squared/df); ^d ^Relative risk (RR) is the effect estimate for the increase in mortality per 1 °F above the threshold temperature; ^e^ Confidence interval (CI).

**Table 5:** Deaths in Maricopa County female residents ages <65 years and ≥65 years directly caused by exposure to environmental heat and other causes potentially related to high temperatures during the months May–October for years 2000–2008.

	Female < 65 Years			Female ≥ 65 Years		
Cause-of-Death Category ^a^	Threshold AT_max _(°F) (Lag Days)^ c^	RR above Threshold ^d^	95% CI^ e^	Threshold AT_max _(°F) (Lag Days)^ c^	RR above Threshold^ d^	95% CI^ e^
Direct exposure to environmental heat	97 (0)	1.19	1.08, 1.31	90 (0)	1.20	1.12, 1.28
Possible consequences of heat or dehydration				105 (0)	1.05	1.01, 1.10
Cardiac disease/stroke	106 (1)	1.05	1.01, 1.09	108 (1)	1.03	1.01, 1.05
Chronic obstructive pulmonary diseases (COPD)/asthma				109 (0)	1.07	1.02, 1.13
All causes ^b^	107 (1)	1.02	1.00, 1.04	107 (1)	1.01	1.00, 1.02

^a ^See Supplemental Material and Supplemental Table S1 for ICD-10 codes and keywords included in cause-of-death categories; ^b ^Excludes deaths from external causes (see Table 2); ^c ^Coefficient for AT_max_ (appropriate lag) has *p*-value < 0.05 in best fitting Poisson regression model (Pearson chi squared/df); ^d ^Relative risk (RR) is the effect estimate for the increase in mortality per 1 °F above the threshold temperature; ^e^ Confidence interval (CI).

Re-estimation of separate mortality models for males and females age <65 and ≥65 showed some similarities and differences in heat-related mortality by gender (Table 4 and Table 5). All groups had relatively low temperature thresholds and high relative risk in the category direct exposure to environmental heat (AT_max_ = 90–97 °F, lag days = 0, RR = 1.19–1.21). The relative risk of death from the possible consequences of heat or dehydration was also elevated in younger males (AT_max_ = 106 °F, RR = 1.18) and older females (AT_max_ = 105 °F, RR = 1.05) with zero lag days. All groups except males ≥65 had elevated relative risk of death from all causes with one-day lag effects. Table 4 shows that, above temperature thresholds, the relative risk of death from respiratory diseases (non-COPD/asthma) was elevated in males age ≥65 and Table 5 shows that the relative risk of death from COPD/asthma was elevated in females age ≥65. Relative risk was elevated for cardiac/stroke deaths in younger females (AT_max_ = 106 °F, RR = 1.05) and older females (AT_max_ = 108 °F, RR = 1.03) with a one-day lag effect.

### 3.2. Discussion

#### 3.2.1. Heat Thresholds and Mortality

We found increasing mortality above threshold temperatures in Maricopa County residents for direct exposure to environmental heat, possible consequences of heat or dehydration, cardiac disease/stroke, respiratory illnesses, and all-cause deaths during May–October for years 2000–2008. Prior heat and health studies in Maricopa County have demonstrated a significant health response to above-normal temperatures, including mortality from heat exposure [38,39] and number of heat-related emergency distress dispatches [40]. A national comparative study of heat wave mortality [8] found that mortality response was lower in southern cities than in temperate cities but the effect in Phoenix was higher than in other southern cities. Yip *et al.* [38] analyzed mortality risk by age, gender, and race in Maricopa County for cause-specific mortality from direct exposure to heat and aggregated cardiovascular/respiratory illnesses. Their findings are consistent with ours in important respects as described in the next few paragraphs. Our results are also consistent with studies of heat-related mortality in many places because deaths associated with high temperatures occurred with very short delays [1,2,6,33,35]. Our study contributes new information on protracted threats from heat mortality in hot climates and the importance of examining different causes of death in specific population groups. 

In Maricopa County, extremely hot weather is an ever-present danger in summer and the number of deaths attributed to heat in Arizona is much higher than in the rest of the U.S. [41]. Although heat-mortality threshold temperatures in Arizona desert cities are much higher than in temperate cities [2,4,9], people died from direct exposure to environmental heat on days below local median seasonal temperatures. In the study period, median daily high AT_max _was 99.5 °F but the heat tolerance threshold was estimated at 93 °F. The NWS advises that prolonged exposure or physical activity requires ‘extreme caution’ when AT (sometimes referred to as the Heat Index) is between 90 °F and 105 °F [42] and our estimated daily AT_max_ thresholds for deaths due to direct heat exposure are at the low end of that range.

Prior research suggests that many of the heat exposure deaths in Maricopa County are due to risk factors that create acute heat stress situations outdoors, such as working in outdoor occupations [38,43], being homeless [38,43,44], and living in low socioeconomic status and hotter neighborhoods [45]. Lack of air conditioning or non-operational units on hot days also contribute to indoor heat deaths [38,46]. Temperature effects on direct heat exposure deaths were statistically significant for all age and gender groups in Maricopa County: younger and older males and females had similarly elevated relative risks above relatively low temperature thresholds in this climate throughout the summer months.

Our results for direct heat exposure deaths were robust and because of their irrefutable connection to heat, they bear closer examination. We identified 215 deaths in residents (average 25.5 deaths/season) in nine summers (2000–2008) using all available information (ICD-10 code and keywords) in death certificates Part I. Mortality in males age <65 outnumbered all other groups: males <65 = 112 deaths (52.1%); females <65 = 22 (10.2%); males ≥65 = 45 (20.9%); females ≥65 = 36 (16.7%). Yip *et al.* [38] reported that the majority of deaths attributed directly to heat during the 2005 heat wave were in white males with median age 55, and the majority of those deaths occurred outdoors. During five summers (2007–2011) of operating a heat surveillance system that uses multiple sources of information to investigate deaths suspected of being associated with environmental heat, the Maricopa County Department of Public Health confirmed 278 heat-associated deaths in residents (average 55.6 deaths/season) [39]. In all likelihood, our study undercounted the number of deaths associated with direct exposure to environmental heat. Studies that use only the cause-of-death ICD-10 code count even fewer cases.

Most literature heavily emphasizes sensitivity of the elderly to environmental heat, especially from cardiovascular and respiratory causes [1,28,32,34,36,47,48]. Heat effects on male and female mortality appear to vary by cause of death and study location [1]. Some studies have not found any gender differences [47,48] but others found the effect of heat mortality was stronger for females [5,36,49,50]. It is important to distinguish among different causes of death in order to understand differences in heat-related mortality between the young and old or females and males. 

The only consistent age difference in our results was elevated relative risk above the temperature thresholds from respiratory illnesses for older males and females. Unlike most prior studies, with the exception of respiratory illnesses, we did not find consistent differences in the heat-related relative risks for other categories of death by age. If fact, our analysis showed that threshold temperatures were lower in people <65 years than in the elderly for cardiac disease/stroke and all-causes deaths. Mortality caused by possible consequences of heat or dehydration had significant relationships with daily AT_max _for younger males and older females. 

The relative risk of death from cardiac disease/stroke above the heat threshold was elevated in younger and older females but not at all in males of any age. We found a significantly different temperature-mortality relationship between males and females for cardiovascular disease, which could suggest gender-specific physiological differences in the effects of sustained hot weather on heat stroke in men and women (e.g., [51]). 

Cardiac disease/stroke deaths are numerically consequential because they alone accounted for 27.4% of all-cause deaths in county residents. The NWS guidelines indicate that heat-related illnesses are likely when AT is in the ‘danger’ zone between 105 and 130 °F [42], and our estimated heat thresholds for death from cardiac disease/stroke, respiratory diseases and other possible consequences of heat and dehydration fall at the lower end of this range between AT_max_ = 105 and 110 °F.

We observed a one-day lag between daily high temperature and cardiac disease/stroke deaths and all-cause deaths. Yip *et al.* [38] also found a significant association of temperature with deaths in a combined cardiovascular/respiratory category. Using time series analysis for the months June–September in 2000–2005 (and focusing especially on a heat wave period in 2005), those authors found an exponential Heat Index-mortality relationship with estimated RR = 1.06 (95% CI: 1.00, 1.13) for each 1 °F increase in temperature. This is in the middle of our estimates for cardiac/stroke, COPD/asthma, and other respiratory diseases (RR = 1.03–1.09) in Table 2 through 5. Yip *et al.* [38] did not estimate thresholds but they reported that cardiovascular/respiratory mortality increased exponentially with temperature as the Heat Index “approaches 100 °F.” Based on the evidence, we conclude that prolonged high temperature during the summer in hot cities is very likely related to higher mortality from cardiovascular disease.

Chronic heat may increase the relative risk of death for people in hot climates because the body’s thermoregulatory response to heat stress includes increased skin blood flow and peripheral blood pooling, which place a burden on the heart to maintain adequate cardiac output and blood pressure. Increased sweating can lead to dehydration and hemoconcentration, which then causes coronary thrombosis and stroke. Our approach to categorizing cardiovascular deaths differs in some respects from the approach taken by others, who have generally done their analyses based on grouping causes of death according to blocks of ICD-10 codes without considering the underlying pathophysiologic mechanism that would link exposure to high environmental temperature and the condition listed as the cause of death. 

We categorized some deaths that are in the “diseases of the circulatory system” block of codes (I00-I99; e.g., deaths due to arrhythmias and sudden death) with deaths in the “endocrine, nutritional and metabolic diseases” block of codes (E00-E90; e.g., hyperosmolality and hypernatremia) because the effect of heat in causing a death attributed to the condition is both direct and immediate and the effect of heat might be expected to be large for these conditions. We did find a lower threshold temperature and a shorter lag for mortality in this “possible consequences of heat and dehydration” category than in our “cardiac disease/stroke” category (Table 2). We analyzed deaths due to heart failure (I50), which are included in the “diseases of the circulatory system” block in most analyses, as a separate cause of death because the additional cardiac burden that accompanies heat stress might be expected to be especially dangerous for this condition. We did not find heart failure mortality to be significantly associated with mortality risk in this setting. 

Several methodologies using time-series techniques have been employed in prior studies to characterize temperature-mortality relationships [2,4,52]. For example, Curriero *et al.* [2] analyzed the temperature-mortality spectrum using relative risk functions for 11 US cities (which did not include Phoenix) during the period 1973–1994. Curriero *et al.*’s analysis yielded approximately J-shaped relative-risk curves and estimated the so-called minimum mortality temperature (MMT) that separates mortality associated with the “cold slope” (temperatures lower than the MMT) from the “hot slope” mortality (temperatures higher than the MMT). Similarly, Muggeo (52) developed a unified model-based approach to identify break points in mortality data as a function of temperature that could indicate increasing physical risk from cold and heat. Muggeo’s approach was used in Milan, Italy, which is characterized by a similar J-shape to those identified by Curriero *et al.* Our statistical method for estimating heat thresholds was adapted from McMichael *et al.*’s [4] ‘ISOTHURM’ study that quantified mortality relationships with cold and heat in a dozen world cities. Due to the absence of a “cold season” in Maricopa County, we focused only on warm months to reduce seasonal fluctuations in mortality. This approach is well suited for settings in hot climates, such as the desert southwestern U.S., where the temperature-mortality relationship includes a substantial temperature interval with a “flat” mortality ratio before a significant mortality increase is observed. It is worth noting that our heat threshold estimates provide the value of temperature at which mortality ratio begins an exponential trend, which should not be confused with the turning points derived in some previous studies. 

In all retrospective studies of temperature-associated mortality, the magnitudes of thresholds for categories of deaths indirectly related to heat must be interpreted with caution. The ISOTHURM study states that threshold estimates can be imprecise when “data are sparse over a very narrow temperature range” [4]. A time series of mortality records longer than nine years would increase the number of observed days that deviate widely from local normal temperatures (Figure 1) and reduce uncertainties around mortality estimates. Figure 2b–i show high variability in mortality on unseasonably cool days (AT_max_ < 78° F). Moreover, high variability in mortality was observed in several death categories (e.g., Figure 2e–h) on extremely hot days. This may not be surprising because relatively few extreme days (e.g., AT_max _> 114 °F, n = 22) occurred during our study period. 

Estimating thresholds for categories of deaths that are not directly attributable to heat is subject to measurement error because a heat effect can be masked by the majority of deaths in that category that are not heat-related. The temperature threshold estimates are much higher for categories of deaths indirectly associated with heat than for deaths directly caused by heat and this may be partly due to overestimating the thresholds for indirect causes. The small numbers of deaths in some cause-of-death categories and population subgroups also warrant caution in interpreting the significance of temperature thresholds and in comparing differences between age and gender groups. There were relatively few deaths in the categories for which we did not identify temperature thresholds (with the exception of direct heat exposure). Consequently, some differences in observed relationships between population subgroups and cause-specific categories of deaths are affected by significant uncertainty associated with reduced statistical power for estimates that were based on small death counts. While the threshold measured for each group may be subject to some measurement error, we did find several temperature effects on deaths in younger people and this should be enough to justify more thorough examinations of the presumption that elderly people are at greater risk of heat-related deaths in hot climates.

Other limitations of this study include the following. Air quality was not measured in our study so we do not know if there are interactions between heat and air pollution. Yip *et al.* [38] found weak associations of heat deaths with PM_10 _and ozone in Maricopa County. As noted in Section 2.2, exposure misclassification is potentially a problem in studies of temperature-related mortality in large urban areas because it is common to use one weather station or to average temperatures over a handful of regional stations. Meteorological measurements at one place may be different than measurements in others at a given time due to the heat island effect, differences in elevation, or wind circulation. The regional temperature-mortality threshold estimates in this study are based on meteorological conditions at the Sky Harbor International Airport station located in the central city. Air temperatures are higher there compared to suburban neighborhoods but not higher than some inner-city neighborhoods and urban fringe areas during the hottest part of the day [24]. There are also different levels of socioeconomic vulnerability to heat stress in populations and neighborhoods throughout this urbanized area [53]. Thus, temperature thresholds for heat-related deaths may be lower in some places and higher in others due to intra-urban differences in physical environments and population characteristics. A recent study used spatial methods to interpolate maximum daily temperature measurements from 19 weather stations in Brisbane, Australia and found that time series models using data from the center-city station and a spatiotemporal model of distributed temperatures performed equally well and provided similar estimates of short-term heat mortality for the whole region [54]. It is beyond the scope of our study to investigate geographic differences in thresholds.

#### 3.2.2. Implications of Climate Change for Future Heat Mortality

Climate projections for central Arizona indicate that future air temperatures will continue to rise in the 21st century due to the effects of global climate change and continuing urban expansion. The North American Regional Climate Change Assessment Program (NARCCAP) provides an ensemble of climate change simulations that couple several regional climate models with atmospheric-ocean general circulation models that are driven by the IPCC SRES A2 scenario [54,56]. Analyzing all NARCCAP model combinations for central Arizona, Grossman-Clarke *et al.* [57] concluded that regional mean daily maximum air temperature in mid-century (averaged for all 2041–2070 summer seasons) is projected to be 110.1 °F. NARCCAP simulations and the Grossman-Clarke *et al.* analysis did not consider the effects of potential urban expansion on future temperature. During the past several decades, however, evening and nighttime temperatures in the city of Phoenix increased relative to rural sites [22]. Georgescu *et al.* [23,58] have demonstrated that projected expansion of urban land uses will induce higher mid-century summer temperatures in the region. Thus, higher summer temperatures are expected in the future because of both global and local forcings, and this could dramatically amplify future heat-related mortality in Arizona.

In the past, humans have adapted physiologically, behaviorally, and technologically to a broad range of temperatures through measures such as better health care and housing, weather warnings, and emergency response [59]. The major technological adaptation to hot weather in the U.S. is the invention and adoption of air conditioning, which accounted for 80% of the substantial national decline in heat-related deaths during the 20th century [60]. Urban mortality studies have shown that air conditioning is an important preventative factor against heat deaths [61]. It is likely that air conditioning has forestalled higher heat-related mortality in central Arizona because its widespread adoption preceded this region’s rapid urbanization in the latter half of the 20th century. Today, 97% of all occupied housing units in the Phoenix metropolitan area have central air compared to 66% of occupied units in the nation [62]. Coverage is 92% in households below the poverty line [62], although the costs of electricity and repairing broken units are barriers to operating air conditioners in many low-income Phoenix households [63]. Control of indoor temperatures appears to have nearly reached the saturation point in this region, leaving accessibility gaps only in low-income households. However, hotter summer weather and continuing drought in the Southwest [64] could increase the potential for regional power failures by increasing demand for electricity and interrupting the water supply to power plants.

Cities in all types of climate regimes are becoming warmer in the 21st century [12,13] and may be subjected to more Arizona-like weather without the benefit of widespread air conditioning that Arizonans use to cope with extreme heat. A recent study asked whether humans will be able to adapt to the future climate in the event that projected levels of global warming would exceed the physiological limit to intolerably high heat stress [65]. Sherwood and Huber [65] maintain that global warming will make some populous areas of the world uninhabitable if the average global temperature rises by at least 7 °C (12.6 °F). Even if future temperatures do not exceed the limit of human physiological tolerance, numerous analyses predict that global heat-related deaths in cities are very likely to increase in the future. High heat mortality in central Arizona, a region that has already made some adaptations to predictable periods of extremely high temperatures, demonstrates that more extensive heat-adaptation plans for climate change are needed in cities worldwide.

## 4. Conclusions

This study found significant temperature-mortality relationships in cities with an extremely hot climate during the warmest season when median daily AT_max _= 99.5 °F. The temperature thresholds, or AT_max_ at which the mortality ratio begins an upward exponential trend varied between a low of AT_max _= 93 °F (zero-day lag) for mortality due to direct exposure to environmental heat to a high of AT_max _= 110 °F (one-day lag) for cardiac disease/stroke. All age and gender subgroups had elevated relative risk above a threshold for direct heat exposure deaths. Females of all ages had elevated relative risk for deaths from cardiac disease/stroke above threshold temperatures. On the hottest days, relative risk was elevated for deaths from respiratory diseases in the elderly and for all-cause deaths in females and younger males. These results suggest it is important to look beyond analyses of all-cause deaths and extreme heat events in order to understand the different consequences of prolonged heat stress for vulnerable populations. More detailed analyses, such as this one, are required to strengthen multi-faceted adaptive measures that address all kinds of heat situations. In view of projected future climate change from global warming and urban expansion, it appears likely that there will be amplified heat-health risks in cities worldwide and managing risks under future conditions will require adaptation planning for chronically high temperatures.

## Supplementary Files

Supplementary File 1 Supplementary Information (PDF, 203 KB)
