# Supplementary material for: Heat-Related Deaths in Hot Cities: Estimates of Human Tolerance to High Temperature Thresholds
Source: Int J Environ Res Public Health. 2014 Mar 20;11(3):3304–26. doi: 10.3390/ijerph110303304 (PMC3987036; doi:10.3390/ijerph110303304)

Supplementary Information

Heat-Related Deaths in Hot Cities: Estimates of Human Tolerance to High Temperature Thresholds

---

Selection of ICD-10 Codes Indicating Conditions and Diseases that Might Be Directly or Indirectly Related to Heat

Diana B. Petitti, M.D., M.P.H.

We selected ICD-10 codes indicating conditions that might be directly or indirectly related to exposure to high environmental temperature based on: (1) knowledge of the direct physiologic effects of exposure to high environmental temperature and the downstream consequences of these physiologic effects on the body; and (2) the results of extensive prior work of others on temperature-mortality relationships.

Eight categories of conditions and diseases used in our analysis are described in more detail below. The first three categories were conditions directly due to exposure to high environmental heat; dehydration (and direct consequence of exposure to high environmental heat); and conditions that are possible consequences of heat exposure and/or dehydration. The last five categories, cardiac disease/stroke, chronic renal failure, heart failure, COPD/asthma, and other respiratory illnesses have been prominent in prior analyses of the association of high environmental heat with death and hospitalization.

Supplemental Table S1 provides a complete list of codes for causes of death in each category and the terms associated with exposure to high environmental heat that are entered as free text in any of the underlying cause of death fields in the first three categories.

Direct Exposure to Environmental Heat

In the human, exposure to high environmental heat is often accompanied by exposure to the sun. Thus, the first set of ICD-10 codes are for conditions that include the terms "sunburn", "sun exposure", and/or "ultraviolet light". These were located by doing a text search of the online version of the ICD-10. The second set of ICD-10 codes identified as being due to the direct effect of heat are those that include the term "heat." These were also located by doing a text search of the online version of the ICD-10.

Dehydration

In the human, exposure to high environmental heat is accompanied by sweating and fluid loss. The consequence of fluid loss is dehydration, which is followed by volume depletion and hypovolemia. ICD-10 codes that included the term "dehydration" were identified by doing a text search of the online version of the ICD-10.

Possible Consequences of Heat and Dehydration

In the human, exposure to high environmental heat is accompanied by sweating and fluid loss. The consequence of fluid loss is dehydration, which is followed by volume depletion and hypovolemia. Supplemental Figure S1 is a graphical depiction of the model that explains our approach to selecting codes considered likely to be due to the physiologic and pathophysiologic consequences of heat and dehydration/volume depletion/hypovolemia.

Volume depletion and hypovolemia can cause hypotension. Hypotension can lead to shock, coma and sudden death. Dehydration/volume depletion/hypovolemia can lead to the formation of kidney stones and thus to renal colic.

Hypovolemia can lead to hemoconcentration and cause disturbances in serum electrolytes (sodium—hypernatremia—and potassium—hyperkalemia) and osmolality (hyperosmolality). The disturbances of serum electrolytes can lead to arrythymias, coma, and sudden death.

Hypovolemia can lead to acute renal failure.

Thirst is a physiologic response to dehydration. When water is consumed in response to thirst without adequate electrolytes, the consequence can be disturbances in serum electrolytes (sodium—hyponatremia—and potassium—hypokalemia) and osmolality (hypo-osmolality).

Disturbances in serum electrolytes can lead to arrythymias, coma, and sudden death.

One consequence of heat and dehydration is hyperthermia, which can directly cause arrhythmias, and can lead to shock, coma, and sudden death. Hyperthermia also can lead to fever and then febrile convulsions. Hyperthermia may cause disseminated intravascular coagulation (DIC).

A number of the consequences of heat and dehydration (e.g., hypotension) can cause malaise and fatigue.

Malaise and fatigue are also symptoms of hyperthermia and also of hypovolemia. “Dizziness and giddiness” is a condition with a specific ICD-10 code that has been identified in other studies as having a relationship to ED visits and hospitalization during heat waves.

We selected the ICD-10 codes that corresponded to the other named conditions that are a consequence of heat and dehydration by reading the ICD-10 code book and using information in the publications of others.

Cardiac Disease/Stroke

Studies of excess mortality and morbidity in heat waves consistently find an association with atherosclerotic heart disease and stroke. The description of the physiologic and pathophysiologic consequences of heat and dehydration provide ample explanation for an effect of heat on mortality and morbidity due to atherosclerotic heart disease and stroke. Hypotension, hemoconcentration, and electrolyte disorders would all be expected to exacerbate the severity of underlying vascular disease and/or lead to coronary or cerebral thrombosis.

We selected the set of ICD-10 codes for atherosclerotic vascular disease that have been used by other researchers noting that the link between hemorrhagic stroke (including subarachnoid hemorrhage) and heat/dehydration and its consequences seems tenuous.

Chronic Renal Failure

Chronic renal failure is associated with a multiplicity of underlying abnormalities of fluid and electrolyte metabolism. Many people with chronic renal failure are being dialyzed. An effect of heat and dehydration and their consequences in exacerbating the severity of chronic renal failure and even perhaps causing death in a person with chronic renal failure has high biologic plausibility. We selected all of the ICD-10 codes for chronic renal failure.

Heart Failure

Heart failure is associated with a multiplicity of underlying abnormalities in handling fluids. People with heart failure are at increased risk of atrial fibrillation and are vulnerable to other cardiac arrhythmias. They are also at increased risk of stroke. An effect of heat and dehydration and their consequences in exacerbating the severity of heart failure or causing death in a person with heart failure has high biologic plausibility.

We selected the ICD-10 codes for heart failure for this category. We also selected the ICD-10 codes for unspecified cardiovascular disease, other and ill-defined heart disease and unspecified heart disease for this category based on the belief that many people who have these non-specific codes listed as a cause of death would have heart failure.

Chronic Obstructive Pulmonary Disease (COPD)/Asthma

Other Respiratory Diseases

Some prior research on deaths and hospitalizations in the context of extreme heat has found strong associations with respiratory illnesses. Following Semenza *et al.* [1] and Schwartz [2], we examined chronic obstructive pulmonary disease (COPD) and asthma separately from infections, influenza, and other respiratory diseases.

References

1. Semenza, J.C.; McCullough, J.E.; Flanders, W.D.; McGeehin, M.A.; Lumpkin, J.R. Excess hospital admissions during the July 1995 heat wave in Chicago. *Am. J. Prev. Med.* **1999**, *16*, 269–277.
2. Schwartz, J. Who is sensitive to extremes of temperature? A case-only analysis. *Epidemiology* **2005**, *16*, 67–72.

Table S1. ICD-10 codes for conditions and diseases directly and indirectly related to high environmental heat.

Direct Exposure to Environmental Heat

ICD-10 [a]	Condition
L55	Sunburn
L55.0	Sunburn of first degree
L55.1	Sunburn of second degree
L55.2	Sunburn of third degree
L55.8	Other sunburn
L55.9	Sunburn, unspecified
L56	Other acute skin changes due to ultraviolet radiation
L56.0	Drug phototoxic response
L56.1	Drug photoallergic response
L56.3	Solar urticaria
L56.4	Polymorphous light eruption
L56.8	Other specified acute skin changes due to ultraviolet radiation
L56.9	Acute skin change due to ultraviolet radiation, unspecified
T67	Effects of heat and light
T67.0	Heatstroke and sunstroke
T67.1	Heat syncope
T67.2	Heat cramp
T67.3	Heat exhaustion, anhydrotic
T67.4	Heat exhaustion due to salt depletion
T67.5	Heat exhaustion, unspecified
T67.6	Heat fatigue, transient
T67.7	Heat oedema
T67.8	Other effects of heat and light
T67.9	Effect of heat and light, unspecified
X30	Exposure to excessive natural heat
X32	Exposure to sunlight

Keywords: [b] Environmental Heat, Excessive Heat, Excessive Natural Heat, Exposure to Heat, Exposure to Heated Environment, Exposure to High Environmental Temperature, Exposure to Hot Desert Environment, Exposure to Hot Environment, Heat Cramps, Heat Effect, Heat Environment, Heat Exhaustion, Heated Environment, Heat Exposure, Heat Related, Heat Stress, Heatstroke, Heat Syncope, High Environmental Temperature, Hiking in Hot Climate, Hot Outside, Sunburn, Sun Exposure, Temperature.

Dehydration

ICD-10 [a]	Condition
E86	Volume depletion
P74.1	Dehydration of newborn
R68.2	Dry mouth, unspecified
X54	Lack of water

Keyword: [b] Dehydration.

Possible Consequences of Heat or Dehydration

ICD-10 [a]	Condition
D65	Disseminated intravascular coagulation [defibrination syndrome]
E87.0	Hyperosmolality and hypernatremia
E87.1	Hypo-osmolality and hyponatremia
E87.4	Mixed disorder of acid-base balance
E87.5	Hyperkalemia
E87.6	Hypokalemia
I46	Cardiac arrest
I46.0	Cardiac arrest with successful resuscitation
I46.1	Sudden cardiac death, so described
I46.9	Cardiac arrest, unspecified
I47	Paroxysmal tachycardia
I47.0	Reentry ventricular arrhythmia
I47.1	Supraventricular tachycardia
I47.2	Ventricular tachycardia
I47.9	Paroxysmal tachycardia, unspecified
I48	Atrial fibrillation and flutter
I49	Other cardiac arrhythmias
I49.0	Ventricular fibrillation and flutter
I49.1	Atrial premature depolarization
I49.2	Junctional premature depolarization
I49.3	Ventricular premature depolarization
I49.4	Other and unspecified premature depolarization
I49.5	Sick sinus syndrome
I49.8	Other specified cardiac arrhythmias
I95.0	Idiopathic hypotension
I95.1	Orthostatic hypotension
I95.8	Other hypotension
I95.9	Hypotension, unspecified
N17	Acute renal failure
N17.0	Acute renal failure with tubular necrosis
N17.1	Acute renal failure with acute cortical necrosis
N17.2	Acute renal failure with medullary necrosis
N17.8	Other acute renal failure
N17.9	Acute renal failure, unspecified
N20	Calculus of kidney and ureter
N20.0	Calculus of kidney
N20.1	Calculus of ureter

N20.2	Calculus of kidney with calculus of ureter
N20.9	Urinary calculus, unspecified
N21	Calculus of lower urinary tract
N21.0	Calculus in bladder
N21.1	Calculus in urethra
N21.8	Other lower urinary tract calculus
N21.9	Calculus of lower urinary tract, unspecified
N23	Unspecified renal colic
P74.2	Disturbances of sodium balance of newborn
P74.3	Disturbances of potassium balance of newborn
P81.0	Environmental hyperthermia of newborn
R00	Abnormalities of heart beat
R00.0	Tachycardia,unspecified
R00.1	Bradycardia,unspecified
R00.2	Palpitations
R00.8	Other and unspecified abnormalities of heart beat
R40.2	Coma, unspecified
R42	Dizziness and giddiness
R50.9	Fever, unspecified
R53	Malaise and fatigue
R55	Syncope and collapse
R56.0	Febrile convulsions
R57.1	Hypovolemic shock
R57.8	Other shock
R57.9	Shock, unspecified
R96	Other sudden death, cause unknown
R96.0	Instantaneous death
R96.1	Death occurring less than 24 hours from onset of symptoms, not otherwise explained
R98	Unattended death

Keywords: [b] Exhaustion, Hyperkalemia, Hypernatremia, Hyperther, Hyperthermia, Hypokalemia, Hyponatremia, Hypovolemia.

Cardiac Disease/Stroke

ICD-10 [a]	Condition
I20	Angina pectoris
I20.0	Unstable angina
I20.1	Angina pectoris with documented spasm
I20.8	Other forms of angina pectoris
I20.9	Angina pectoris, unspecified
I21	Acute myocardial infarction
I21.0	Acute transmural myocardial infarction of anterior wall
I21.1	Acute transmural myocardial infarction of inferior wall
I21.2	Acute transmural myocardial infarction of other sites
I21.3	Acute transmural myocardial infarction of unspecified site
I21.4	Acute subendocardial myocardial infarction
I21.9	Acute myocardial infarction, unspecified
I22	Subsequent myocardial infarction
I22.0	Subsequent myocardial infarction of anterior wall

I22.1	Subsequent myocardial infarction of inferior wall
I22.8	Subsequent myocardial infarction of other sites
I22.9	Subsequent myocardial infarction of unspecified site
I24	Other acute ischemic heart diseases
I24.1	Dressler's syndrome
I24.8	Other forms of acute ischemic heart disease
I24.9	Acute ischemic heart disease, unspecified
I25	Chronic ischemic heart disease
I25.0	Atherosclerotic cardiovascular disease, so described
I25.1	Atherosclerotic heart disease
I25.2	Old myocardial infarction
I25.3	Aneurysm of heart
I25.4	Coronary artery aneurysm
I25.5	Ischemic cardiomyopathy
I25.6	Silent myocardial ischemia
I25.8	Other forms of chronic ischemic heart disease
I25.9	Chronic ischemic heart disease, unspecified
I49.9	Cardiac arrhythmia, unspecified
I60	Subarachnoid hemorrhage
I60.0	Subarachnoid hemorrhage from carotid siphon and bifurcation
I60.1	Subarachnoid hemorrhage from middle cerebral artery
I60.2	Subarachnoid hemorrhage from anterior communicating artery
I60.3	Subarachnoid hemorrhage from posterior communicating artery
I60.4	Subarachnoid hemorrhage from basilar artery
I60.5	Subarachnoid hemorrhage from vertebral artery
I60.6	Subarachnoid hemorrhage from other intracranial arteries
I60.7	Subarachnoid hemorrhage from intracranial artery, unspecified
I60.8	Other subarachnoid hemorrhage
I60.9	Subarachnoid hemorrhage, unspecified
I61	Intracerebral hemorrhage
I61.0	Intracerebral hemorrhage in hemisphere, subcortical
I61.1	Intracerebral hemorrhage in hemisphere, cortical
I61.2	Intracerebral hemorrhage in hemisphere, unspecified
I61.3	Intracerebral hemorrhage in brain stem
I61.4	Intracerebral hemorrhage in cerebellum
I61.5	Intracerebral hemorrhage, intraventricular
I61.6	Intracerebral hemorrhage, multiple localized
I61.8	Other intracerebral hemorrhage
I61.9	Intracerebral hemorrhage, unspecified
I62	Other nontraumatic intracranial hemorrhage
I62.0	Subdural hemorrhage (acute) (nontraumatic)
I62.1	Nontraumatic extradural hemorrhage
I62.9	Intracranial hemorrhage (nontraumatic), unspecified
I63	Cerebral infarction
I63.0	Cerebral infarction due to thrombosis of precerebral arteries
I63.1	Cerebral infarction due to embolism of precerebral arteries
I63.2	Cerebral infarction due to unspecified occlusion or stenosis of precerebral arteries
I63.3	Cerebral infarction due to thrombosis of cerebral arteries
I63.4	Cerebral infarction due to embolism of cerebral arteries

I63.5	Cerebral infarction due to unspecified occlusion or stenosis of cerebral arteries
I63.6	Cerebral infarction due to cerebral venous thrombosis, nonpyogenic
I63.8	Other cerebral infarction
I63.9	Cerebral infarction, unspecified
I64	Stroke, not specified as hemorrhage or infarction
I67	Other cerebrovascular diseases
I67.0	Dissection of cerebral arteries, nonruptured
I67.1	Cerebral aneurysm, nonruptured
I67.2	Cerebral atherosclerosis
I67.3	Progressive vascular leukoencephalopathy
I67.4	Hypertensive encephalopathy
I67.5	Moyamoya disease
I67.6	Nonpyogenic thrombosis of intracranial venous system
I67.7	Cerebral arteritis, not elsewhere classified
I67.8	Other specified cerebrovascular diseases
I67.9	Cerebrovascular disease, unspecified
I69	Sequelae of cerebrovascular disease
I69.0	Sequelae of subarachnoid hemorrhage
I69.1	Sequelae of intracerebral hemorrhage
I69.2	Sequelae of other nontraumatic intracranial hemorrhage
I69.3	Sequelae of cerebral infarction
I69.4	Sequelae of stroke, not specified as hemorrhage or infarction
I69.8	Sequelae of other and unspecified cerebrovascular diseases
I70	Atherosclerosis
I70.0	Atherosclerosis of aorta
I70.1	Atherosclerosis of renal artery
I70.2	Atherosclerosis of arteries of the extremities
I70.8	Atherosclerosis of other arteries
I70.9	Generalized and unspecified atherosclerosis

Chronic Renal Failure

ICD-10 [a]	Condition
N18	Chronic renal failure
N18.0	End-stage renal disease
N18.8	Other chronic renal failure
N18.9	Chronic renal failure, unspecified
N19	Unspecified renal failure

Heart Failure

ICD-10 [a]	Condition
I50	Heart failure
I50.0	Congestive heart failure
I50.1	Left ventricular failure
I50.9	Heart failure, unspecified
I51.6	Cardiovascular disease, unspecified
I51.8	Other ill-defined heart diseases
I51.9	Heart disease, unspecified

Chronic Obstructive Pulmonary Diseases (COPD)/Asthma

ICD-10 [a]	Condition
J43	Emphysema
J43.0	MacLeod syndrome
J43.1	Panlobular emphysema
J43.2	Centrilobular emphysema
J43.8	Other emphysema
J43.9	Emphysema, unspecified
J44	Other COPD
J44.0	COPD with acute lower respiratory infection
J44.1	COPD with acute exacerbation, unspecified
J44.8	Other specified COPD
J44.9	COPD, unspecified
J45	Asthma
J45.0	Predominantly allergic asthma
J45.1	Nonallergic asthma
J45.8	Mixed asthma
J45.9	Asthma, unspecified
J46	Status asthmaticus

Other Respiratory Diseases

ICD-10 [a]	Condition
J00-J06	Acute upper respiratory infections
J09-J18	Influenza and pneumonia
J20-22	Other acute lower respiratory infections
J30-39	Other diseases of the upper respiratory tract
J40-J42	Bronchitis
J47	Bronchiectasis
J60-J70	Lung diseases due to external agents
J80-J84	Other respiratory diseases principally affecting the interstitium
J85-J86	Suppurative and necrotic conditions of lower respiratory tract
J90-J94	Other diseases of pleura
J95-J99	Other diseases of the respiratory system

[a] International Classification of Diseases, 10th Revision. [b] We searched the database for selected keyword terms entered as free text in any of the four underlying cause-of-death fields in Part I of the death certificate. Cases with keywords were included in one of the first three categories of death (*i.e.*, direct exposure to environmental heat, dehydration, or possible consequences of heat and dehydration). Cases with keywords associated with more than one of these categories were assigned to the category most directly associated with heat; *i.e.*, a case with a keyword from direct exposure to environmental heat and a keyword from possible consequences of heat and dehydration, was assigned to the first category.

Figure S1. Approach to selection of ICD-10 codes for conditions that are likely due to physiologic and pathophysiologic consequences of heat and dehydration.

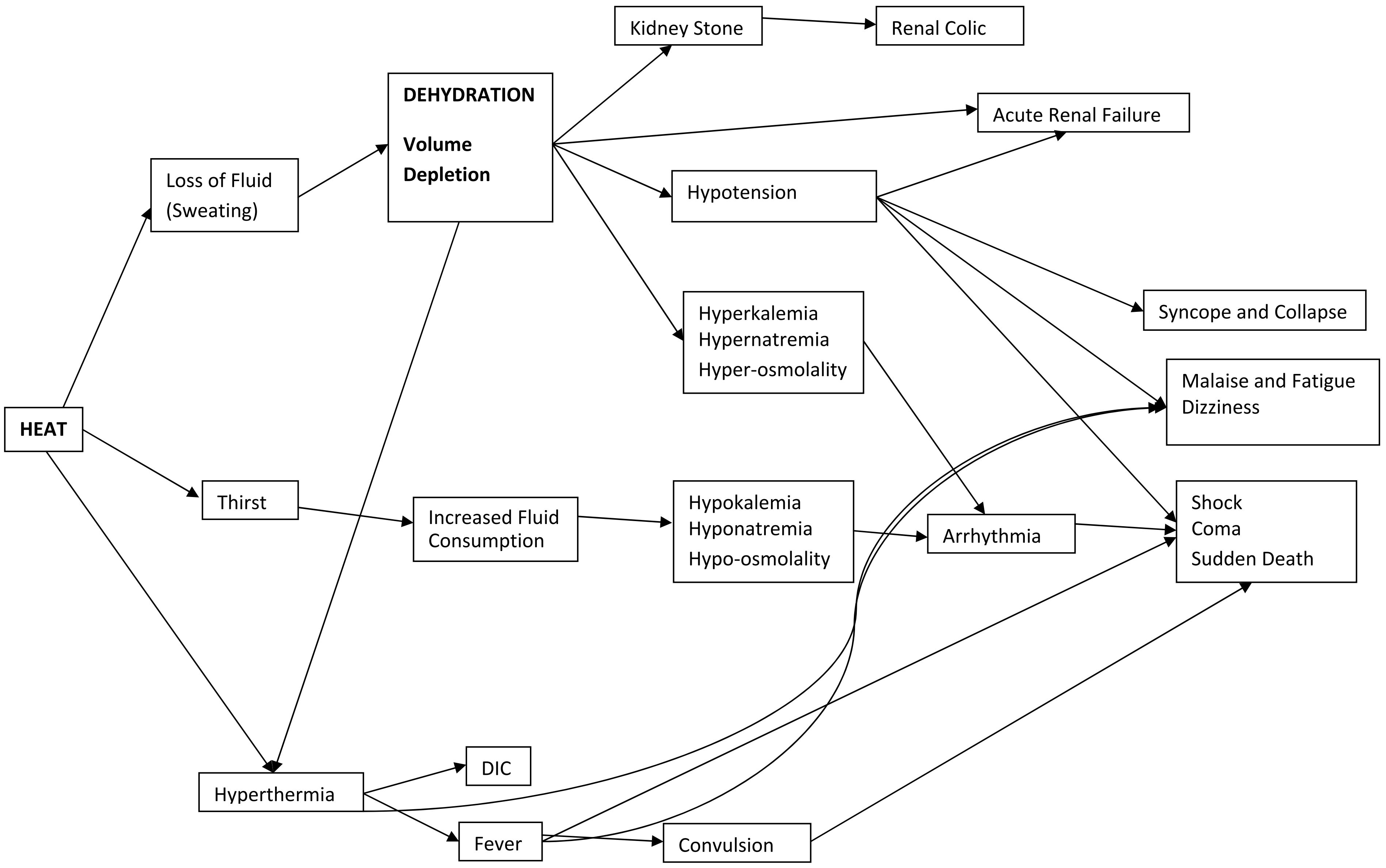

Supplement: Supplementary File 1 — Supplementary Information (PDF, 203 KB) [file ijerph-11-03304-s001.pdf]
